# Supplementary figures and images for: XA4C: eXplainable representation learning via Autoencoders revealing Critical genes
Source: PLoS Comput Biol. 2023 Oct 2;19(10):e1011476. doi: 10.1371/journal.pcbi.1011476 (PMC10569512; doi:10.1371/journal.pcbi.1011476)

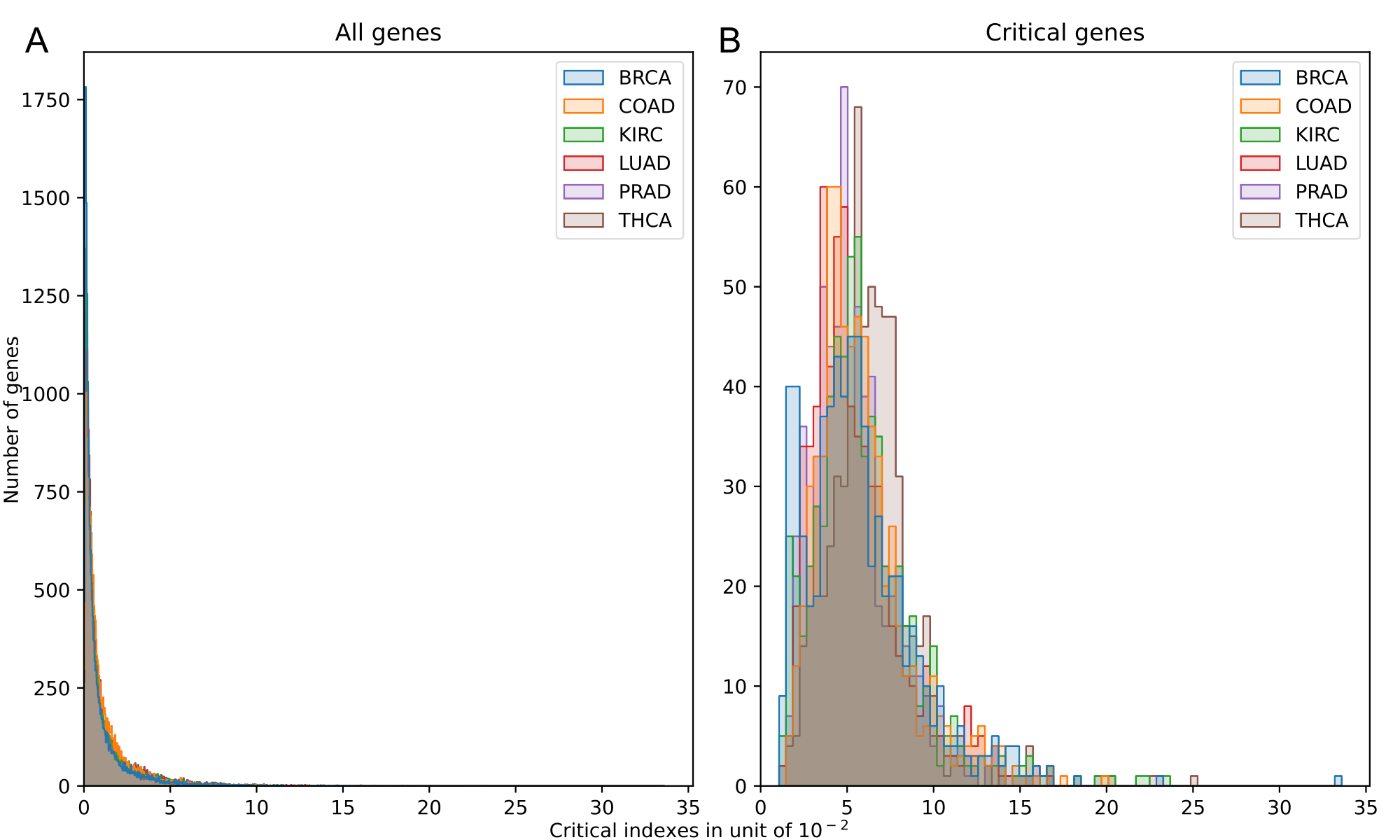

Supplement: S1 Fig — (A) Distribution of pathway Critical indexes for all genes in the corresponding pathways. (B) Distribution of pathway Critical indexes for Critical genes in the corresponding pathways. (TIF) [file pcbi.1011476.s001.tif]
