# Supplementary material for: XA4C: eXplainable representation learning via Autoencoders revealing Critical genes
Source: PLoS Comput Biol. 2023 Oct 2;19(10):e1011476. doi: 10.1371/journal.pcbi.1011476 (PMC10569512; doi:10.1371/journal.pcbi.1011476)
Supplement: Attachment — Submitted filename: ResponseLetter_20230723.pdf [file pcbi.1011476.s012.pdf]

Reviewer #1: Review Gen

This paper addresses the challenge of interpreting learned representations from autoencoders in gene expression analyses. It introduces a novel tool called XA4C (eXplainable Autoencoder for Critical genes), which combines state-of-the-art techniques in eXplainable Artificial Intelligence (XAI) with autoencoders. XA4C employs optimized autoencoders at global and local levels to process gene expressions and utilizes SHapley Additive exPlanations (SHAP) to quantify the contribution of individual genes to the learned latent variables.

Introduction
Overall, the introduction section provides a clear background on the significance of ML models in gene expression analysis, addresses the limitations of existing approaches, and introduces the XA4C tool as a solution for explainable analysis and prioritization of critical genes which is good.

To highlight the novelty of XA4C, it would be helpful to explicitly state how it differs from existing interpretable ML tools and what unique features or capabilities it brings to the field. This will help readers understand the specific contributions of XA4C.

Thanks for the overall positive evaluation. In our initial submission, such comparisons were presented in Discussion. In the revised manuscript, based on your comment, we have integrated them into Introduction.

Result
When conducting pathway over-representation analysis, it would be valuable to include statistical significance measures, such as p-values or false discovery rates, to determine the significance of pathway enrichment. This would provide more robust evidence for the involvement of specific pathways in cancer.

Thank you for the constructive comments, and we agree that p-values are robust evidence to show the involvement of particular pathways in cancer. Actually, we had presented adjusted p-values at a false discovery rate of 0.05 using the colors of dots in enrichment analysis in Figure 3A (red for small values and blue for large ones), although the actual values were not included. In the revised manuscript, we included the detailed adjusted p-values at a false discovery rate of 0.05 in Supplementary S2 Table. A sentence has been added to the caption of Fig 3 to refer readers to the table for detailed values.

General
In order to adhere to the standard practice of writing abbreviations, the author should provide the full name or description of an abbreviation the first time it is mentioned, followed by the abbreviation in parentheses. However, for subsequent mentions of the same abbreviation within the same section or context, it is generally not necessary to repeat the full name or description. Instead, the abbreviation can be used directly.
To address the issue in the provided lines, the author should modify the text as follows:
Line 120 variables. To quantify each gene’s contribution to the latent variables, XA4C employs eXtreme Gradient Boosting (XGBoost)
Line 339: eXtreme Gradient Boosting 19 (XGBoost) Regresso

Line 377: pathway representations and their corresponding inputs were passed through the eXtreme Gradient Boosting (XGBoost)

Thank you for pointing this out. We have removed the duplicated definition of XGBoost and also proofread other places to fix similar problems.

Reviewer #2:

Tha manuscript proposed “Critical genes”, defined as genes that contribute highly to learned representations. Then, applied eXplainable Autoencoder on the genes to find netowrk of genes and highly contributing genes (discriminative genes) for each type of studied cancer (BRCA, etc..). The manuscript is wll-presented, the methods are properly applied. However, I have some minor concerns:

- The literature lack of the recent application of explainableAI in cancer and health outcomes. KI suggest if the authors may highlight studies such as (PMID: 36738712 and/or PMID: 37233630).

Thanks for providing missing essential literature. We have cited these in the revised manuscript (in Introduction, the end of page 4).

-The results does not show any performance measurements such as accuracy, sensitivity, etc.

-AUCROC curve of the prediction model may shows the performance of the model.

Thank you for your valuable comments. In the revised manuscript, we have added a section to present performance measurements to evaluate the performance of our XA4C model in comparison to the hub genes and DiffEx genes. The outcomes show that Critical genes have better performance than alternatives (Supplementary S6 and S7 Tables). We have added the description of this analysis in Results (Page 15 Line 266-270). The related method is also detailed in Materials & Methods, subsection “Performance measurements of accuracy and sensitivity” (Page 22-23, Line 466- 480). For the reviewer’s convenience, we outline the methods and results below:

In this evaluation, we first construct confusion matrices. Considering DisGeNET-reported genes as the gold-standard. For a particular tool (critical genes, hub genes, or DiffEx genes), we defined true positives (TP) as genes identified by the tool and are reported by DisGeNET, true negatives (TN) as genes not identified and not reported in DisGeNET, false positives (FP) as genes identified but not reported in DisGeNET, and false negatives (FN) as genes identified but not reported in DisGeNET. Based on the confusion matrices (Supplementary S6 Table), we calculated precision, recall, F1 score and accuracy (detailed formulation defined in Materials & Methods).

We compared the performance of XA4C Critical genes to hub genes and DiffEx genes. The results, presented in Supplementary S7 Table, demonstrate that XA4C outperforms the other methods in terms of the F1-score in all six cancers, and these three methods have similar performance in terms of accuracy.

It should be noted that, in practice, when people use Hub gene or DiffEx gene analyses, they usually use a fixed parameter, i.e., the default setting, without optimizing outcomes using a tuning parameter. In our study, as stated in Materials & Methods, we utilized the "chooseTopHubInEachModule" function from the Weighted Correlation Network Analysis (WGCNA) package. We applied this function to the gene expression matrix obtained from pathways, while maintaining the default settings for other parameters. Specifically, we set the power parameter to 2 and the type parameter to "signed." As for DiffDESeq2, it employs a generalized linear model framework with a negative binomial distribution to assess differential expression between two groups. Initially, it estimates the fold change for each gene between the groups, and subsequently calculates the Wald test statistics and corresponding p-values. These p-values reflect the level of evidence contradicting the null hypothesis that there is no disparity in gene expression between the conditions. These p-values are further adjusted for multi-test correction, and the significance level (alpha) utilized is 0.05, a conventional parameter for statistical tests. Analogously, we designed Critical genes generated by XA4C as the genes that are in the top 1% among all genes contributing to the autoencoder analysis.

Therefore, all these tools (hub genes and DiffEx genes and Critical genes) will not face the trade-offs of adjusting tuning parameters in practice. As such, the AUCROC curve may not be the most suitable measure for evaluating their relative performance. The above analysis of F1-score etc., on default parameters has reflected the quantitative performance measurement of accuracy suggested by the reviewer.

Also how the model avoided over-fitting.

Thanks for reminding us of the issue of over-fitting. We have added a paragraph addressing all issues related to overfitting in Discussion in the revised manuscript (Page 17, Line 312 – 322):

Machine learning algorithms may run into overfitting. In XA4C, there are two models used: Autoencoder and TreeSHAP. The autoencoder by itself is unsupervised, therefore, it may not run into overfitting [1, 2]. More importantly, a sparsity penalty with L1 regularization is applied to XA4C autoencoder loss function, which penalizes non-zero activations. This sparsity penalty can prevent overfitting to some extent because it makes the autoencoder prefer to activate only a subset of its nodes. It also helps generalization by preventing the model from remembering noisy or irrelevant patterns in the training data [3, 4]. It is important to note that TreeSHAP itself does not introduce overfitting if the underlying tree model is not overfitting. In our study, we employed the XGBoost regression model as the tree model. XGBoost models also incorporate regularization techniques to prevent overfitting [5, 6]. With the regularization penalty in both the autoencoder and TreeSHAP, we believe the overfitting is under control in our XA4C model.

References
1. Michelucci U. An introduction to autoencoders. arXiv. 2022.
2. Lorbeer B, Botler M. Anomaly Detection with Partitioning Overfitting Autoencoder Ensembles. Proc Spie. 2022;12084. doi: 10.1117/12.2622453. PubMed PMID: WOS:000799214600003.
3. Zhang CF, Cheng X, Liu JH, He J, Liu GW. Deep Sparse Autoencoder for Feature Extraction and Diagnosis of Locomotive Adhesion Status. J Control Sci Eng. 2018;2018. doi: 10.1155/2018/8676387. PubMed PMID: WOS:000440514100001.
4. Meng LH, Ding SF, Xue Y. Research on denoising sparse autoencoder. Int J Mach Learn Cyb. 2017;8(5):1719-29. doi: 10.1007/s13042-016-0550-y. PubMed PMID: WOS:000408104000025.

5. Chen TQ, Guestrin C. XGBoost: A Scalable Tree Boosting System. Kdd'16: Proceedings of the 22nd Acm Sigkdd International Conference on Knowledge Discovery and Data Mining. 2016:785-94. doi: 10.1145/2939672.2939785. PubMed PMID: WOS:000485529800092.
6. Gomez-Rios A, Luengo J, Herrera F. A Study on the Noise Label Influence in Boosting Algorithms: AdaBoost, GBM and XGBoost. Hybrid Artificial Intelligent Systems, Hais 2017. 2017;10334:268-80. doi: 10.1007/978-3-319-59650-1_23. PubMed PMID: WOS:000432880600023.

Reviewer #3: XA4C: A Tool for the Identification of Critical Genes in Cancer

Summary
Li et al.'s work is of considerable importance in cancer genomics. Identifying critical genes associated with various types of cancer is crucial for understanding the mechanisms underlying the disease and developing effective therapeutic strategies. The authors have combined autoencoders and the SHAP framework to develop a new computational model, XA4C, which is aimed at extracting hidden features from transcriptome data and determining the contribution of each gene. Integrating these advanced machine learning techniques allows for a more comprehensive analysis and understanding of high-dimensional gene expression data. The ability of XA4C to uncover novel critical genes could potentially contribute to early detection, personalised treatment strategies, and new insights into the biology of cancer.

Thank you for the thorough summary and positive evaluation.

To enhance the value of this work, the manuscript should incorporate comparisons with existing models, integrates a thorough methodology for gene identification by considering multiple genetic alterations and validate the results with established databases. These additions would enrich the manuscript's quality and fortify its relevance in cancer research.

Thank you for the constructive comments. We have thoroughly revised the manuscript based on your input. Please see our item-to-item response below.

Comments
1. Consider Multiple Criteria for Identifying Cancer-Related Genes: The manuscript emphasises the use of differential expression in identifying critical genes. However, it is important to acknowledge that differential expression and hub genes are not the only criteria for determining cancer genes. There are various factors, such as changes in DNA methylation, gain-of-function mutations in oncogenes, loss-of-function mutations in tumour suppressor genes, copy number alterations, chromatin accessibility, and changes in protein expression, that also play a role in cancer progression. The authors could enhance the manuscript by analysing whether the critical genes identified through the XA4C model are associated with some or any of these changes in the studied cancer types. This broader approach can provide a more comprehensive understanding of the genes' roles in cancer.

Thank you for the comments. In order to analyze whether the Critical genes identified through the XA4C model are associated with some or any of these changes in the studied cancer types, we have resorted to COSMIC database to check whether the XA4C-identified genes are indeed in overlap with genes with the genetic (or epigenetic) mutations mentioned by the reviewer. We first obtained information from the COSMIC database: the genetic mutations, including missense mutations and copy number variations, and epigenetic mutations, including differential

methylation. Based on the available mutation information, we observed a significant proportion of Critical genes (70% averaged over six cancers) that exhibited gained or lost copy number variations. Additionally, approximately 25% of the Critical genes showed differential methylation, characterized by a beta-value difference larger than 0.5 compared to the average beta-value across the normal population. Furthermore, around 12% of the Critical genes displayed missense mutations, which have the potential to alter the function of the encoded proteins. In the revised manuscript, the detailed results have been added as Supplementary S4 Table, and have been presented in Results (Page 13 Line 224-234).

2. Validate Findings with Known Cancer Genes from COSMIC Database: To increase the robustness and credibility of the findings, the authors should consider validating the critical genes identified by the XA4C model against known cancer genes listed in the COSMIC (Catalogue of Somatic Mutations in Cancer) database. By evaluating which among the identified critical genes are classified as Tier 1 and Tier 2 cancer genes in relation to the differentially expressed genes and the hub genes, the authors can provide additional evidence that supports the utility and accuracy of the XA4C model in identifying relevant cancer genes. This validation with a reputable external database would add significant value and trustworthiness to the results presented in the manuscript.

Thanks for the suggestion. In order to validate the Critical genes identified by the XA4C model, as well as the hub genes and DiffEx genes, we compared them using the COSMIC database's census genes (both Tier 1 and Tier 2) associated with specific cancers. There are 738 genes presented in the COSMIC cancer census. However, only 200 genes are specific to the six cancers analyzed in our study (Supplementary S5 Table). Although we observed only a small overlap between the Critical genes and these census genes, the overlap ratios are comparatively higher than the overlaps observed between census genes and Hub genes or DiffEx genes for genes in (Supplementary S5 Table). This demonstrates consistency with the results obtained from analyzing the enrichment of genes using the DisGeNET database. We have added this outcome to the revised manuscript (Page 15, Line 261-265).

3. Incorporate Comparisons with Existing Models: The manuscript presents the XA4C model, which combines autoencoders and SHAP values to interpret the contributions of individual genes in the context of cancer transcriptome data. It might be beneficial for the authors to include a comparison section where the performance and interpretability of XA4C are rigorously compared to other existing models and techniques in the same field. This will help in validating the robustness and utility of the XA4C model. This could include traditional statistical methods such as XGBoost or Random Forests approaches.

Thanks for the comments. We performed a comparison between the Critical genes identified by XA4C and the genes prioritized by Random Forest and XGBoost by using the "feature importance values" generated by Random Forest and XGBoost classifiers, respectively. The detailed procedure is in Materials & Methods (Page 23, Line 485-499), and the outcome is presented in Discussion (Page 17-18, Line 326-334). For the reviewer's convenience, we also outline the procedure and outcomes here:

The Random Forest and XGBoost classifiers were trained on gene expressions from 335 pathways. The classifiers were trained using default parameter settings with 500 estimators (number of trees in the forest). To ensure a balanced representation of tumor and normal

samples, we randomly sampled tumor samples with the same number of normal tissue samples to construct datasets. The resulting dataset was then divided into training and test datasets in a 7:3 ratio (Supplementary S8 Table). We performed model optimization on training datasets and evaluated its performance on test datasets. Notably, the classifiers exhibited impressive performance, as indicated by high weighted-averaged F1 scores across the 335 pathways. Specifically, the Random Forest classifier achieved an F1 score of 94% for six cancers, while the XGBoost classifier achieved an F1 score of 92% for the same six cancers (Supplementary S8 Table).

Subsequently, feature importance values were derived from these well-trained classifiers. Similar to the identification of Critical genes, we defined genes with top 1% ceiling importance values from classifiers in each pathway as "Important genes" (Supplementary S9 Table).

Remarkably, when annotating the genes using the same databases (DisGeNET and COSMIC), the Important genes identified by Random Forest or XGBoost exhibited a lower enrichment in the DisGeNET databases when compared to the critical genes (Supplementary S10 Table). The same trend of XA4C's advantage is also observed in the COSMIC database.

Overall, thank you so much for the constructive comments, which significantly strengthened our manuscript.
